# Characterization and Expression Analysis of Heme Oxygenase Genes from *Sorghum bicolor*

**DOI:** 10.1177/1177932219860813

**Published:** 2019-07-12

**Authors:** Takalani Mulaudzi-Masuku, Vivian Ikebudu, Mpho Muthevhuli, Andrew Faro, Christoph A Gehring, Emmanuel Iwuoha

**Affiliations:** 1 Department of Biotechnology, University of the Western Cape, Bellville, South Africa; 2 Department of Chemistry, Biology & Biotechnology, University of Perugia, Perugia, Italy; 3 SensorLab, Department of Chemistry, University of the Western Cape, Bellville, South Africa

**Keywords:** Heme oxygenase, HO expression, osmotic stress, quantitative real-time polymerase chain reaction, signature motif, *Sorghum bicolor*

## Abstract

Heme oxygenases (HOs) have a major role in phytochrome chromophore biosynthesis, and chromophores in turn have anti-oxidant properties. Plant heme oxygenases are divided into the HO1 sub-family comprising HO1, HO3, and HO4, and the HO2 sub-family, which consists of 1 member, HO2. This study identified and characterized 4 heme oxygenase members from *Sorghum bicolor*. Multiple sequence alignments showed that the heme oxygenase signature motif (QAFICHFYNI/V) is conserved across all *Sb*HO proteins and that they share above 90% sequence identity with other cereals. Quantitative real-time polymerase chain reaction revealed that *Sb*HO genes were expressed in leaves, stems, and roots, but most importantly their transcript level was induced by osmotic stress, indicating that they might play a role in stress responses. These findings will strengthen our understanding of the role of heme oxygenases in plant stress responses and may contribute to the development of stress tolerant crops.

## Introduction

Abiotic stresses especially water deficit and salinity are the leading factors affecting plant growth and development and thus reduction in crop productivity especially in rain-fed areas.^1–3^ These factors are the major causes of osmotic stress, which results in turgor loss due to low water availability and accumulation of excess Na and Cl ions.^4^ Turgor pressure is maintained through osmotic regulation, and this is important for plant growth by cell expansion.^5,6^ Significant changes in water potentials in the environment can impose osmotic stress to plants, resulting in various physiological changes, such as excessive production of reactive oxygen species (ROS), which further causes oxidative damage to cells, loss of membrane function, enzyme inactivation, DNA and protein denaturation, as well as ionic and nutrient imbalance.^7^ Plants have evolved several mechanisms to respond to osmotic stress, and these include changes in their life cycle, adjustment of ion transport, synthesis of compatible solutes, and the detoxification of ROS through the anti-oxidative system.^8^ The anti-oxidative system is divided into the non-enzymatic and enzymatic components, in which the latter is more effective consisting of enzymes such as superoxide dismutase (SOD), catalase (CAT), ascorbate peroxidase (APX), glutathione peroxidase (GPX), and glutathione reductase (GR).^9,10^ In addition, the heme oxygenase-1 (HO1) enzyme system has also gained more attention due to its anti-oxidative properties.^11–15^ Heme oxygenase (EC 1.14.99.3) catalyzes the formation of biliverdin-IXα (BV), carbon monoxide (CO), and free iron (Fe^2+^) through the oxidation of heme.^16,17^ Biliverdin-IXα is further converted into bilirubin (BR) by biliverdin reductase.^18^ Biliverdin-IXα and BR possess strong anti-oxidant properties in mammals^19,20^ and plants,^16^ whereas CO is well recognized as a strong anti-oxidant that regulates ROS homeostasis in animals.^14^ These statements support the idea that HO1 may play a protective role against oxidative damage.

Plant genomes encode 4 HO genes, namely, HO1, HO2, HO3, and HO4, which were initially identified in the model species, *Arabidopsis thaliana*. Heme oxygenases include a small gene family with 2 main sub-families including the HO1 sub-family which consists of 3 members, namely, HO1, HO3, and HO4, whereas the HO2 sub-family has only 1 member, HO2.^21,22^ Members are differentially expressed with HO1 representing the most highly expressed, followed by HO2, while both HO3 and HO4 are expressed at low levels.^21^ In general, HO1 is known to provide all the anti-oxidative protective effects that are associated with HOs. It is induced by various stimuli, including heavy metals,^23–26^ NO,^27,28^ glutathione depletion,^29^ UV radiation,^30^ heme, paraquat,^31^ H_2_O_2_,^32,33^ and salinity.^11,15,34,35^ HO2 is also induced by NO, H_2_O_2_,^12^ hemin, paraquat, and salinity.^21,22,36^ The expression and induction of HO genes in response to stress represent their role in mediating a defense mechanism against various stresses.

To date, plant HO genes have only been identified and characterized in a few species including *A. thaliana* (*At*HO1-*At*HO4),^37^ *Oryza sativa* (*Os*HO1 and *Os*HO2),^36,38^ *Medicago sativa* (*Ms*HO1 and *Ms*HO2),^12^ *Brassica oleracea* (*Bo*HO1),^25^ *Triticum aestivum* (*Ta*HO1),^39^ *Brassica napus* (*Bn*HO1 and *Bn*HO3),^40^ *Zea mays* (*Zm*HO1),^41^ *Nicotiana tabacum* (*Nt*HO1),^42^ and *Glycine max* (*Gm*HO1 and *Gm*HO3).^43,44^ Some of the challenges in the identification and characterization of HO genes in plants has been attributed to the lack of the publicly available genomic sequences, for example, in wheat.^39^ Sorghum (*Sorghum bicolor*) is one of the most important cereal crops worldwide and is considered to be moderately tolerant to drought and salinity.^45^ In sorghum, the genome sequence is available^46,47^ and HO genes (*Sb*HO1 and *Sb*HO2) were previously sequenced^44^ while the other two putative members, *Sb*HO3 and *Sb*HO4, are only annotated. In this study, bioinformatic approaches were employed to identify and characterize HO genes in *Sorghum bicolor*. The sequence parameters of all four sorghum HO genes (*Sb*HO1-*Sb*HO4) including gene structure, physicochemical properties, subcellular localization, signature motifs, and evolutionary relationship were analyzed and compared with other HOs from different plant species. Finally, their gene expression profiles in response to osmotic stress were also elucidated using quantitative real-time polymerase chain reaction (qRT-PCR). Our results provide novel insights into the structure, evolution, and the expression profiles of sorghum HOs, particularly in response to osmotic stress.

## Materials and Methods

### Plant growth and treatment

Red sorghum (*Sorghum bicolor*) seeds purchased from Agricol, Brackenfell, South Africa, were germinated as described previously^48^ with slight modification. Briefly, plant tissue culture vessels containing half strength Murashige and Skoog (MS) media (2.2 g/L MS; 1% [w/v] sucrose; 5 mM MES; and 0.4% [w/v] agar; pH 5.8) and 4 seeds per vessel were incubated at 25°C under a 16-h light/8 h dark regime for 14 days prior to mannitol treatment. For stress treatments, seedlings were carefully transferred to half strength MS media supplemented with 250 mM mannitol and incubated for 3, 12, and 24 h under the same growth conditions. Untreated seedlings were used for the control samples (0 h). After stress treatments, tissues that were separated into leaves, stems, and roots were immediately frozen in liquid nitrogen and stored at −80°C until further use.

### Sequence retrieval

Four *Sorghum bicolor* HO sequences representing HO1 (*Sb*HO1; AAK63010.1), HO2 (*Sb*HO2; AAK63011.1), HO3 (*Sb*HO3; XP_002438642.1), and HO4 (*Sb*HO4; XP_021304790.1) were obtained in FASTA format from the National Center for Biotechnology Information (NCBI) protein database (http://www.ncbi.nlm.nih.gov/). For comparative purposes, sequences of orthologous genes were obtained from the Phytozomev.10.3^49^ and NCBI databases to represent a proteome dataset of 32 selected plant species. These include the following: *Asparagus officinalis, Aegilops tauschii, Amborella trichopoda, A. thaliana, Brachypodium distachyon, Brassica juncea, Brassica napus, Chenopodium quinoa, Cucumis sativus, Cucurbita maxima, Cucurbita moschata, Dendrobium catenatum, Elaeis guineensis, Glycine max, Gossypium hirsutum, Hevea brasiliensis, Hordeum vulgare, Jatropha curcas, Manihot esculenta, Medicago sativa, Nicotiana tabacum, Oryza sativa, Phalaenopsis equestris, Sesamum indicum, Setaria italica, Solanum lycopersicum, Solanum tuberosum, Sorghum bicolor, Spinacia oleracea, Triticum aestivum, Zea mays*, and *Ziziphus jujuba*.

### Prediction of gene structure and physicochemical parameters

The Gene Structure Display Server online tool (GSDS 2.0)^50^ was used to analyze the exon-intron structure of HO genes by comparing with the CDS sequences and genomic DNA sequences. Properties such as protein length, molecular weight (*M_w_*) and theoretical isoelectric point (*pI*) were computed using the ExPASy Proteomic server.^51^ Subcellular localization of the different proteins was predicted by CELLO.^52^

### Prediction of conserved domains and signature motifs

Protein sequences were aligned using the ClustalW2 program of the European Bioinformatics Institute.^53^ To deduce the protein family and explore the domain arrangement within proteins, sequences were analyzed using the Conserved Domain Database (CDD)^54^ while prediction of conserved motifs was performed using the Multiple Expectation Maximization for Motif Elicitation (MEME 5.0.2) online tools. Parameter settings for MEME were as follows: maximum number of motifs to find: 5; minimum width of motif: 6; and maximum width of motif: 50.^55^

### Phylogenetic analysis

To determine the evolutionary relationships, phylogenetic analysis was performed using Molecular Evolutionary Genetics Analysis version 7.0 (MEGA 7) for bigger database.^56^ The program was used to generate a boot-strapped dataset of 1000 replicates. The pair-wise deletion and *p*-distance model by neighbor-joining (NJ) methods were used.

### Total RNA extraction and reverse transcriptions

Total RNA was extracted from 100 mg of 2-week-old sorghum seedlings (leaves, stems, and roots) using the Favorgen plant mini RNA extraction kit (Favorgen Biotech Corp., Ping-Tung, Taiwan) according to the manufacturer’s instructions. The RNA was treated with the RNase-free DNase reaction Set (New England Biolabs, Massachusetts) to remove genomic DNA and its quality was determined by analyzing on a 1% agarose gel. Concentration and purity were determined using a NanoDrop spectrophotometer (Thermo Scientific, USA). About 1 µg of the total RNA was used for first-strand cDNA synthesis using the SuperScript™ III First-Strand Synthesis kit (Invitrogen, Carlsbad, California, USA) according to the manufacturer’s instructions.

### Quantitative real-time PCR

Quantitative real-time PCR (qRT-PCR) was used to analyze the tissue-specific expression profiles of *Sb*HO genes. The reaction mixture contained 1 µL template cDNA, 5 µL 2× SYBR Green I Master Mix (Roche Applied Science, Germany), varying concentrations of each primer and ddH_2_O added to a final volume of 10 µL. The reactions were subjected to 95°C for 10 min, 45 cycles at 95°C for 10 s, 55°C for 10 s, and 72°C for 20 s. A melting curve analysis was also performed using default parameters on the LightCycler^®^ 480 instrument (Roche Applied Science, Germany). The primer information of the target genes (*Sb*HOs) and the reference genes (ubiquitin [UBQ] and phosphoenolpyruvate carboxylase [PEPC]) is shown in Table 1. The expression levels of the target genes were normalized to the reference genes and analyzed using the LightCycler^®^ 480 SW (version 1.5) data analysis software. The expression was quantified by relative quantification method using a standard curve of serially diluted cDNA templates. Each qRT-PCR reaction was done in triplicate and 3 non-template controls were included. Each experiment represent an average of 3 independent experiments. Figures were plotted using Microsoft Excel (2013). cDNA amplicons of the *Sb*HO genes which were amplified by qRT-PCR to produce 202 bp (*Sb*HO1), 250 bp (*Sb*HO2), 202 bp (*Sb*HO3), and 215 bp (*Sb*HO4) fragments were all sequenced (Supplementary Figure 1).

**Table 1.** Names of the genes and their accession numbers used for designing primers used in the quantitative real-time PCR experiment.

Gene name	Forward primer	Reverse primer	Accession number
*Sb*HO1	5′-TTCCAGACGCTCGAAGACAT –3′	5′-CCTGGGGATCCTTCTCAGAC –3′	AF320026.1
*Sb*HO2	5′-GGAAAAGTGGTTTGGAGCGT –3′	5′- AACTCCAGCTCCCTTCCTTC-3′	AF320027.1
*Sb*HO3	5′-TTCCAGACGCTCGAAGACAT-3′	5′-CCTGGGGATCCTTCTCAGAC-3′	XM_002438597.2
*Sb*HO4	5′-TTCCTCGTCGATAGCAAGCT-3′	5′-TTCCCAGACAGCTCTTCCAG-3′	XM_021449115.1
UBQ	5′-GCC AAG ATT CAG GAT AAG –3′	5′-TTG TAA TCA GCC AAT GTG –3′	XM_002452660
PEPC	5′-GAA GAA TAT CGG CAT CAA T-3′	5′-CTA TGT AAT ACT TGG TAA CTT TC-3′	XM_002438476

## Results and Discussion

### Sequence comparison and analysis of physicochemical properties

Plant HOs are mainly known for their involvement in the biosynthetic pathway for the production of phytochrome chromophores that is important for photomorphogenesis.^37^ HOs also participate in plant growth and development,^41,57,58^ and their role in protecting cells against oxidative stress is well documented.^17,23,59^ Heme oxygenases have been annotated in many plant species but their functional characterization is still limited. This study was undertaken to characterize the HO genes from sorghum in comparison with other plant species using bioinformatics tools and analyze their gene expression profiles using qRT-PCR toward identifying their potential biological function. Heme oxygenase members were identified by searching the NCBI database using the BLAST tool and a total of 43 HO orthologs from 32 plant species were obtained. Among the 43 HO orthologs, 4 belonged to sorghum and these include the *Sb*HO1 (accession numbers: AAK63010.1, AF320026.1), *Sb*HO2 (accession numbers: AAK63011.1, AF320027.1), HO1, chloroplastic *Sorghum bicolor* (accession numbers: XP_002438642.1, XM_002438597.2; gene ID: Loc8065066, Sobic.3010G184600), and HO1, chloroplastic *Sorghum bicolor* (accession numbers: XP_021304790.1, XM_021449115.1; Gene ID: Loc8065071, Sobic.3010G184800). The two putative HO1 candidate genes will be referred to as *Sb*HO3 and *Sb*HO4, respectively, according to their position on chromosome 10. Heme oxygenase genes were further confirmed for the presence of the conserved heme oxygenase domain (PF01126) using the Pfam database and the CDD tool indicating that the sorghum HO proteins are likely to perform the same function as other known and characterized plant HOs.

**Figure 1.**
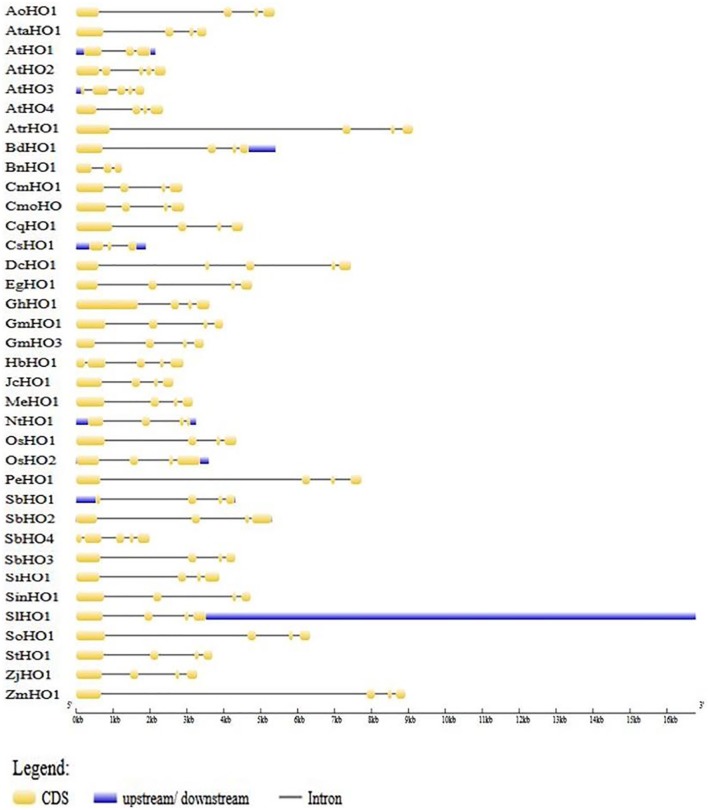
Exon-intron organization of heme oxygenase genes from different plant species. Genes analyzed are those from the species listed in Table 2. Species used for this analysis include the following: *Asparagus officinalis* (*Ao*HO1), *Arabidopsis thaliana* (*At*HO1, *At*HO2, *At*HO3, *At*HO4), *Aegilops tauschii* (*Ata*HO1), *Amborella trichopoda (Atr*HO1), *Brachypodium distachyon* (*Bd*HO1), *Brassica napus* (*Bn*HO1), *Cucurbita maxima* (*Cm*HO1), *Cucurbita moschata* (*Cmo*HO1), *Chenopodium quinoa* (*Cq*HO1), *Dendrobium catenatum* (*Dc*HO1), *Elaeis guineensis* (*Eg*HO1), *Glycine max* (*Gm*HO1, *Gm*HO3) *Gossypium hirsutum* (*Gh*HO1), *Hevea brasiliensis* (*Hb*HO1), *Jatropha curcas* (*Jc*HO1), *Manihot esculenta* (*Me*HO1), *Nicotiana tabacum* (*Nt*HO1), *Oryza sativa* (*Os*HO1, *Os*HO2), *Phalaenopsis equestris* (*Pe*HO1), *Sorghum bicolor* (*Sb*HO1, *Sb*HO2, *Sb*HO3, *Sb*HO4), *Setaria italica* (*Si*HO1), *Sesamum indicum* (*Sin*HO1), *Solanum lycopersicum* (*Sl*HO1), *Spinacia oleracea* (*So*HO1), *Solanum tuberosum* (*St*HO1), *Ziziphus jujuba* (*Zj*HO1), *Zea mays* (*Zm*HO1), and *Cucumis sativus* (*Cs*HO1).

Characteristics of HOs, including the gene ID, physicochemical parameters, and localization are shown in Table 2. To understand the gene structure of sorghum HOs, genomic sequences and their corresponding CDS sequences were retrieved and analyzed using the GSDS online tool. The structural diversity of different plant HO genes was obtained, analyzed, and showed that 36 HO orthologs have 3 to 5 exons, with most HOs (28 in total) having 4 exons; 5 HOs have 5 exons, and 3 HOs have 3 exons (Figure 1). To be more specific, the first 2 exons of *Sb*HO1, 2 and 3, are interrupted by long introns. *Sb*HO4 displayed a unique gene structure from the rest of the *Sb*HO genes, since it has 5 exons which are interrupted by short introns. The CDS lengths varied from 282 to 1381 bp and encoded polypeptides of 184 to 338 amino acid residues. The molecular weight (*M_w_*) of the different polypeptides ranged from 21.3 to 37.06 kDa with *pI* values of 5.39 to 9.19. Three *Sb*HO proteins, namely, *Sb*HO1, *Sb*HO2, and *Sb*HO3, are characterized by a *pI* that is less than 7 suggesting that they are acidic, while *Sb*HO4 (*pI* = 8.58) is basic. Another difference is that the *Sb*HO4 gene encodes a longer polypeptide with a *M_w_* (37.6 kDa) that is slightly higher than the other *Sb*HO proteins. These data suggest that *Sb*HO4 is structurally diverse and might perform a unique function. Subcellular localization of the HO proteins was predicted to be in the chloroplast, nucleus, mitochondria, and cytoplasm (Table 2), and these data correlate with previous studies.^59^ These results indicate that sorghum HO candidates share some similarities with other plant HOs.

**Table 2.** List of heme oxygenase homologs from 32 plant species and their physiochemical parameters.

Species name	Phytozome gene ID	CDS (bp)	Exon no.	Protein length (aa)	MW (KDa)	*pI*	Localization CELLOᵇ
*Sorghum bicolor* HO1	Sobic.3010G184600	557	4	184	21.3	5.59	Cyto
*Sorghum bicolor* HO2	Sobic.3001G347800	987	4	328	36.43	5.39	Nucl/Chlo
*Sorghum bicolor* HO4	Sobic.3010G184800	1017	5	338	37.06	8.58	Chlo
*Sorghum bicolor* HO3	Sobic.3010G184600	867	4	288	31.88	6.42	Chlo
*Aegilops tauschii* HO1	LOC109775269	849	4	288	31.63	6.21	Chlo
*Amborella trichopoda* HO1	AMTR_s00078p00062760	876	4	305	34.89	9.19	Mito/Chlo
*Arabidopsis thaliana* HO1	AT2G26670	849	3	282	32.69	6.50	Cyto/Chlo
*Arabidopsis thaliana* HO2	AT2G26550	900	5	299	34.90	5.80	Nucl
*Arabidopsis thaliana* HO3	AT1G69720	684	5	227	25.60	8.24	Mito
*Arabidopsis thaliana* HO4	AT1G58300	852	4	283	32.95	6.98	Cyto
*Asparagus officinalis* HO1	LOC109819496	846	4	272	31.11	8.88	Mito/Cyto
*Brassica juncea* HO1	−	849	−	282	32.62	7.68	Chlo
*Brassica juncea* HO2	−	699	−	232	26.40	6.97	Nucl
*Brassica juncea* HO3	−	846	−	281	32.27	6.99	Cyto
*Brassica napus* HO1	Brara.G01278	282	3	282	32.62	8.27	Chlo/Cyto
*Brachypodium distachyon* HO1	Bradi1g36640	870	4	279	31.08	7.03	Chlo/Mito
*Chenopodium quinoa* HO1	AUR62011131-RA	870	4	280	32.18	8.72	Cyto/Mito
*Cucumis sativus* HO1	−	1381	4	291	33.31	7.67	Chlo
*Cucurbita maxima* HO1	LOC111500260	819	4	297	33.92	8.30	Chlo
*Cucurbita moschata* HO1	LOC111441000	918	4	297	34.02	8.61	Chlo
*Dendrobium catenatum* HO1	LOC110104187	867	5	285	32.41	8.74	Mito/Cyto
*Elaeis guineensis* HO1	LOC105040158	876	4	282	32.19	8.95	Cyto
*Glycine max* HO1	GLYMA_04GG147700	282	4	282	32.11	8.63	Cyto
*Glycine max* HO3	GLYMA_06G221900	282	4	282	32.07	8.83	Cyto
*Gossypium hirsutum* HO1	LOC107962617	858	4	285	32.96	7.71	Mito/Cyto
*Hevea brasiliensis* HO1	LOC110648797	837	5	291	33.62	8.44	Cyto/Mito
*Hordeum vulgare* HO1		867	−	288	31.62	6.41	Chlo
*Jatropha curcas* HO1	LOC105639364	837	4	291	33.29	7.70	Chlo
*Manihot esculenta* HO1	Manes.14G132400	858	4	289	33.30	8.72	Cyto/Chlo
*Medicago sativa* HO1	−	852	−	283	32.62	6.21	Cyto
*Medicago sativa* HO2	−	870	−	290	33.59	8.67	Nucl
*Nicotiana tabacum* HO1	LOC107818591	819	4	278	32.27	8.50	Mito/Cyto
*Oryza sativa* HO1	LOCOs6G40080	1262	4	289	31.91	6.28	Chlo
*Oryza sativa* HO2	LOCOs3G27770	282	4	330	36.54	4.92	Chlo
*Phalaenopsis equestris* HO1	LOC110022387	894	4	285	32.53	7.64	Cyto
*Sesamum indicum* HO1	LOC105162092	882	4	272	31.18	7.73	Cyto
*Setaria italica* HO1	Seita.4G223200	840	4	282	31.44	6.14	Chlo/Mito
*Solanum lycopersicum* HO1	Solyc12g009470	993	4	278	31.96	6.98	Cyto/Mito
*Solanum tuberosum* HO1	LOC102600014	849	4	278	32.00	7.66	Cyto/Mito
*Spinacia oleracea* HO1	LOC110776560	837	4	281	32.18	8.26	Cyto/Mito
*Triticum aestivum* HO1	−	867	−	288	31.65	6.41	Chlo
*Zea mays* HO1	GRMZM2G101004	1262	4	285	31.60	6.63	Chlo
*Ziziphus jujuba* HO1	LOC107434821	849	4	293	33.43	8.81	Mito/Cyto

Abbreviation: MW, molecular weight.

“–” indicates that the genomic sequence and/or gene ID was not available.

### Prediction of signature and conserved motifs

**Figure 2.**
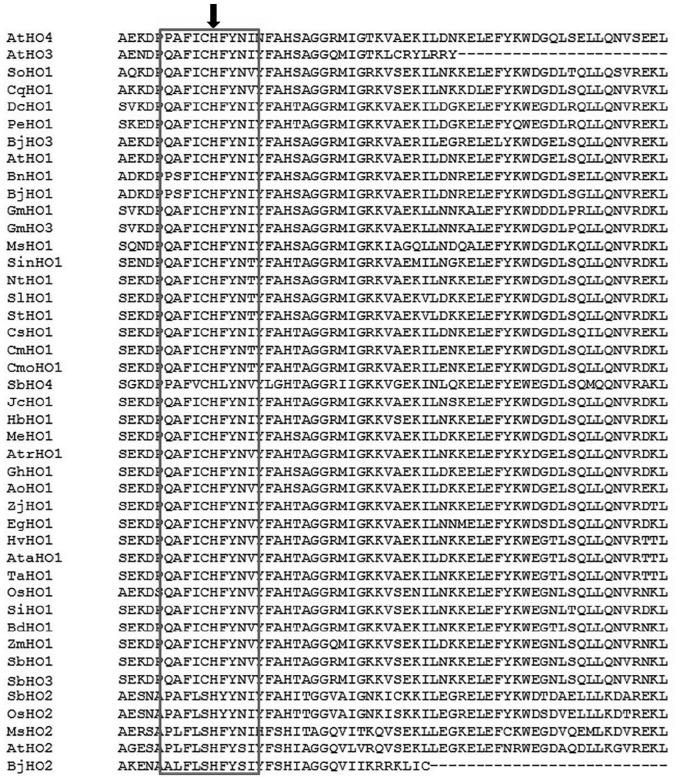
Multiple sequence alignment of *Sb*HO genes and other HO orthologs in plant species. The conserved HO amino acids are emphasized with a red block. The black arrow pointing downward indicates the conserved histidine residue for protein stability. Species included in this analysis are the same as those analyzed in the gene structure with addition to *Medicago sativa* (*Ms*HO1 and *Ms*HO2), *Brassica juncea* (*Bj*HO1, *Bj*HO2, and *Bj*HO3), *Triticum aestivum* (*Ta*HO1), and *Hordeum vulgare* (*Hv*HO1).

Sequence signature motifs are generally conserved in a protein family since they perform similar structural and functional roles,^60^ thus it is important to consider them while grouping novel proteins within specific families. The presence of the HO signature motif was analyzed by multiple sequence alignment using the ClustalX software based on the amino acid sequences of each HO protein. Based on the alignment, almost all amino acid sequences of the HO proteins were conserved in the HO signature sequence (QAFICHFYNI/V), which is important for heme binding (Figure 2). Slight sequence variations within the signature motif were observed within some HO proteins, including *At*HO4 (PAFICHFYNIN), *Sb*HO4 (PAFVCHLYNV) and *Bn*HO1 (PSFICHFYNI). *Sb*HO2 and *Os*HO2 share an identical signature sequence (PAFLSHYYNI), which is different from *At*HO2 (PLFLSHFYSIYF). *Sb*HO1, *Sb*HO2, *Sb*HO3, and *Sb*HO4 share 72%, 53%, 70%, and 51% amino acid sequence identity with *At*HO1, *At*HO2, *At*HO3, and *At*HO4, respectively. In addition, *Sb*HOs share above 90% sequence identity with HOs from cereals suggesting a high level of functional similarities.

**Figure 3.**
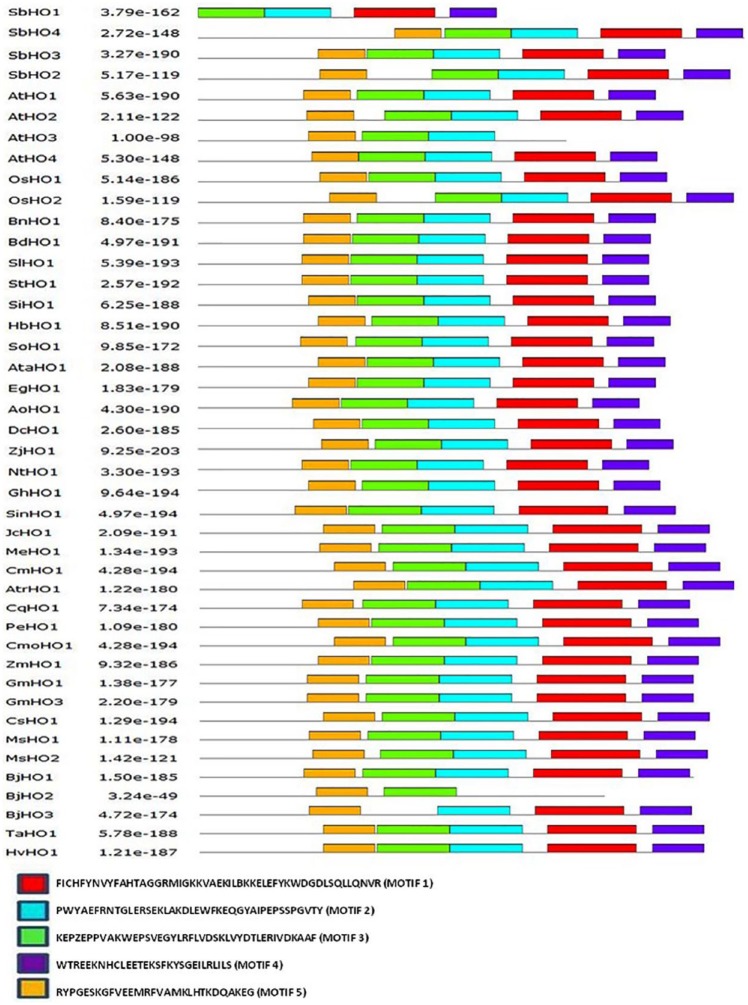
Conserved motifs of the heme oxygenase proteins. Different motifs are shown by different colored boxes. Species included in this analysis are the same as those analyzed in the multiple sequence alignment.

Conserved HO motifs were predicted using the online MEME tool to better understand the protein’s evolution and function. A total of 5 motifs were predicted and their sequence verified using BLAST (Figure 3). Only motif 1 and motif 2 encoded the HO super-family and are present in all the HO orthologs except for *At*HO3 and *Bj*HO2. Motifs 3, 4, and 5 did not encode any conserved domain. Since motif 1 and 2 were found in all HO proteins including the 4 sorghum HOs, the results provide confidence of their identification as *bona fide* HO encoding genes and infer a functional similarity with other HOs.

### Phylogenetic analysis

**Figure 4.**
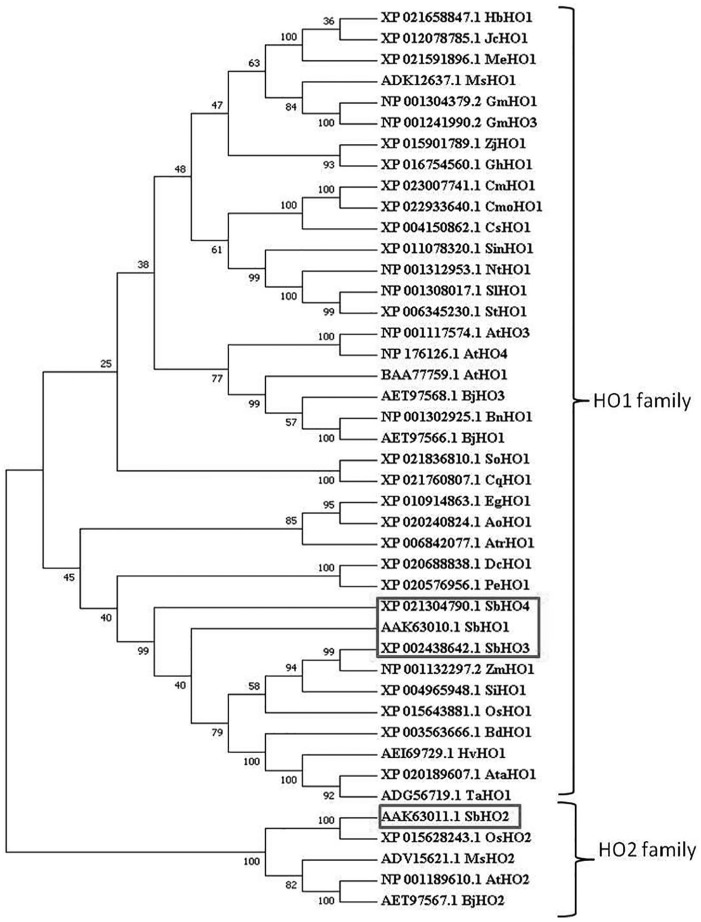
Phylogenetic tree showing evolutionary relationships between *Sb*HO sequences and HOs from other plant. The amino acid sequences of *Sb*HOs were aligned with 39 HO orthologs from other plant species using ClustalW2 and analyzed using MEGA 7 program by neighbor-joining method with 1000 bootstrap replicate.

Phylogenetic relationships of HOs from sorghum were compared with other known and well-characterized HO members from other plant species. The phylogenetic tree was constructed using the neighbor joining method, based on the sequence alignment of 43 full-length HO amino acid sequences from 32 plant species to examine the conservation and diversity of the HO domain region (Figure 4). The tree comprises 2 main classes (class I and II); both of which are divided into 2 main groups with several branches. Class I is the biggest class comprising all HO1 members including the sorghum HO1 candidates, whereas class II comprises only HO2s from sorghum, rice, mustard, alfalfa, and Arabidopsis (Figure 4). Based on the phylogenetic analysis, *Sb*HO1, *Sb*HO3, and *Sb*HO4 can be grouped together as belonging to the HO1 sub-family since they fall in class I. These results are consistent with previous reports,^39,42^ and they indicate that the HO family is highly conserved across plant species, with close sequence correlation of *Sb*HOs observed with other cereals.

### Analysis of the expression profiles of SbHO genes

**Figure 5.**
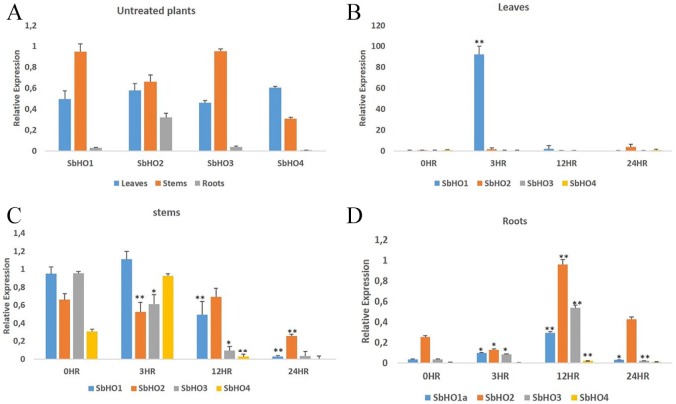
Expression analysis of *Sorghum bicolor* HO gene family under osmotic stress. Seedlings grown in MS media in the absence (0 h) and presence of 250 mM mannitol to induce osmotic stress at different time points of 3, 12, and 24 h. (A) Expression analysis of *Sb*HO genes in different tissues, under normal conditions. Comparative expression analysis of *Sb*HO genes in (B) leaves, (C) stems, and (D) roots grown under osmotic stress. Error bars represent the SD calculated from 3 biological replicates and significance differences between control and treated plants were determined using *t-test* shown as **P ⩽ .01 and *P ⩽ .05.

To analyze the expression profiles of *Sb*HO genes (*Sb*HO1, *Sb*HO2, *Sb*HO3, and *Sb*HO4), qRT-PCR was performed on different tissues of sorghum seedlings. *Sb*HO transcripts were expressed in all tissues including leaves, stems, and roots but their level of expression was different under normal conditions (Figure 5A). *Sb*HO genes displayed the same pattern of transcript levels in all tissues with the highest observed in the stems, followed by leaves and roots. The *Sb*HO4 transcript was more expressed in the leaves as compared with the other *Sb*HO members. Based on these expression profiles, all 4 *Sb*HO genes are constitutively expressed in all tissues, which suggest that they might be required for growth and development of plants under normal conditions.

The expression of *Sb*HO genes was analyzed in sorghum seedlings treated with 250 mM mannitol to induce osmotic and hence oxidative stress at different short time-points of 3, 12, and 24 h using qRT-PCR. The analysis indicated that *Sb*HO transcripts were differentially expressed in all tissues and at different time-points (Figure 5B-D). A significant (*P* ⩽ .01) increase in transcript level was observed for *Sb*HO1 in the leaves at 3 h showing a 100-fold increase compared to the control (Figure 5B). While no significant increase was observed in the stem (Figure 5C), a slight increase in the roots (Figure 5D) at 3 and 12 h was observed as compared with the control. Overall, a significantly high *Sb*HO1 transcript level was observed in the leaves upon stress treatment. Upon stress treatment, *Sb*HO2 transcripts were increased in the leaves at 3 and 24 h compared to the control (0 h), showing a 3- and 6-fold increase, respectively. While not much change was observed in the stem, *Sb*HO2 transcripts significantly (*P* ⩽ .01) increased in the roots at 12 h showing ~2-fold increase compared with the control. Similar to the expression pattern of *Os*HO2,^36^ our results indicated that *Sb*HO2 was also significantly induced by osmotic and oxidative stress. The *Sb*HO3 transcript was slightly downregulated in the leaves and stem but a significant (*P* ⩽ .01) increase in the roots at 12 h was observed showing a 12-fold increase as compared with the control (Figure 5D). The *Sb*HO4 transcript was slightly induced in the leaves at 24 h, followed by a 3-fold increase in the stem at 3 h and a slight increase was observed in the roots at 12 and 24 h.

In comparison, significant expression levels were observed for *Sb*HO1 transcript in the leaves followed by *Sb*HO2, *Sb*HO4, and *Sb*HO3, while in the roots, *Sb*HO2 is the most expressed followed by *Sb*HO3 and *Sb*HO1. These results are consistent with previously published data on *A. thaliana* HO members^21^ which suggest that HO genes are differentially expressed and their expression is dependent on the induction by oxidative stress.^15,61^ The model plant “*A. thaliana*” is the only plant species where all four HO members have been identified and characterized. These results revealed that the expression of *Sb*HO genes is differentially regulated by osmotic and oxidative stress in different tissues and at different time-points, indicating that individual members have specific spatial and temporal functions. Since *Sb*HO1 and *Sb*HO2 were previously sequenced, this study provided evidence that the two additional putative HO candidates (*Sb*HO3 and *Sb*HO4) exist and that all four members potentially might play a protective role in sorghum against oxidative stress.

## Conclusion

This study identified and characterized all 4 HO genes in sorghum and their expression in response to osmotic stress analyzed. Results revealed that based on gene structure, subcellular localization, signature motifs, and phylogenetic analysis of HO family members are highly conserved across all plant species analyzed. The data indicated that *Sb*HO genes are transcriptionally expressed in all tissues tested, and expression analysis confirmed that they are inducible by osmotic stress. For future research, it will be interesting and valuable to generate transgenic crops overexpressing sorghum HOs toward understanding their biological role under different stresses. Thus, the study has added new information regarding the possible role of sorghum HOs as part of the defense systems against osmotic and oxidative stress and these data might be useful in the development of stress-tolerant crops.

## Supplemental Material

Supplementary_Figure_1_xyz19475d220d457 – Supplemental material for Characterization and Expression Analysis of Heme Oxygenase Genes from *Sorghum bicolor* Click here for additional data file. Supplemental material, Supplementary_Figure_1_xyz19475d220d457 for Characterization and Expression Analysis of Heme Oxygenase Genes from *Sorghum bicolor* by Takalani Mulaudzi-Masuku, Vivian Ikebudu, Mpho Muthevhuli, Andrew Faro, Christoph A Gehring and Emmanuel Iwuoha in Bioinformatics and Biology Insights

## References

[1] Chaves MM Oliveira MM. Mechanisms underlying plant resilience to water deficits: prospects for water-saving agriculture. J Exp Bot. 2004;55:2365–2384. doi:10.1093/jxb/erh269. 15475377

[2] Kijne JW. Abiotic stress and water scarcity: identifying and resolving conflicts from plant level to global level. F Crop Res. 2006;97:3–18.

[3] Barnabas B Jager K Feher A. The effect of drought and heat stress on reproductive processes in cereals. Plant Cell Environ. 2008;31:11–38. doi:10.1111/j.1365-3040.2007.01727.x. 17971069

[4] Binzel ML Hess FD Bressan RA Hasegawa PM. Intracellular compartmentation of ions in salt adapted tobacco cells. Plant Physiol. 1988;86:607–614. doi:10.1104/pp.86.2.607. 16665954 PMC1054531

[5] Maggio A Zhu JK Hasegawa PM Bressan RA. Osmogenetics: Aristotle to Arabidopsis. Plant Cell. 2006;18:1542–1557. doi:10.1105/tpc.105.040501. 16809814 PMC1488915

[6] Zonia L Munnik T. Life under pressure: hydrostatic pressure in cell growth and function. Trends Plant Sci. 2007;12:90–97. doi:10.1016/j.tplants.2007.01.006. 17293155

[7] Hussain HA Hussain S Khaliq A et al Chilling and drought stresses in crop plants: implications, cross talk, and potential management opportunities. Front Plant Sci. 2018;9:393. doi:10.3389/fpls.2018.00393. 29692787 PMC5902779

[8] Xiong L Zhu JK. Molecular and genetic aspects of plant responses to osmotic stress. Plant Cell Environ. 2002;25:131–139. 11841658 10.1046/j.1365-3040.2002.00782.x

[9] Apel K Hirt H. Reactive oxygen species: metabolism, oxidative stress, and signal transduction. Annu Rev Plant Biol. 2004;55:373–399. doi:10.1146/annurev.arplant.55.031903.141701. 15377225

[10] Farooq M Aziz T Basra SMA Cheema MA Rehman H. Chilling tolerance in hybrid maize induced by seed priming with salicylic acid. J Agron Crop Sci. 2008;194:161–168. doi:10.1111/j.1439-037X.2008.00300.x.

[11] Xie Y Ling T Han Y et al Carbon monoxide enhances salt tolerance by nitric oxide-mediated maintenance of ion homeostasis and up-regulation of antioxidant defence in wheat seedling roots. Plant Cell Environ. 2008;31:1864–1881. doi:10.1111/j.1365-3040.2008.01888.x. 18811735

[12] Fu GQ Xu S Xie YJ et al Molecular cloning, characterization, and expression of an alfalfa (Medicago sativa L.) heme oxygenase-1 gene, MsHO1, which is pro-oxidants-regulated. Plant Physiol Biochem. 2011;49:792–799. doi:10.1016/j.plaphy.2011.01.018. 21316255

[13] Liu Y Xu S Ling T Xu L Shen W. Heme oxygenase/carbon monoxide system participates in regulating wheat seed germination under osmotic stress involving the nitric oxide pathway. J Plant Physiol. 2010;167:1371–1379. doi:10.1016/j.jplph.2010.05.021. 20674075

[14] Piantadosi CA. Carbon monoxide, reactive oxygen signaling, and oxidative stress. Free Radic Biol Med. 2008;45:562–569. doi:10.1016/j.freeradbiomed.2008.05.013. 18549826 PMC2570053

[15] Zilli CG Balestrasse KB Yannarelli GG Polizio AH Santa-Cruz DM Tomaro ML. Heme oxygenase up-regulation under salt stress protects nitrogen metabolism in nodules of soybean plants. Environ Exp Bot. 2008;64:83–89.

[16] Shekhawat GS Verma K. Haem oxygenase (HO): an overlooked enzyme of plant metabolism and defence. J Exper Botany 2010;61:2255–2270. doi:10.1093/jxb/erq074. 20378668

[17] He H He L. Heme oxygenase 1 and abiotic stresses in plants. Acta Physiol Plant. 2014;36:581–588. doi:10.1007/s11738-013-1444-1.

[18] Gozzelino R Jeney V Soares MP. Mechanisms of cell protection by heme oxygenase–1. Annu Rev Pharmacol Toxicol. 2010;50:323–354. doi:10.1146/annurev.pharmtox.010909.105600. 20055707

[19] Stocker R Yamamoto Y McDonagh AF Glazer AN Ames BN. Bilirubin is an antioxidant of possible physiological importance. Science. 1987;235:1043–1046. 3029864 10.1126/science.3029864

[20] Llesuy SF Tomaro ML. Heme oxygenase and oxidative stress: evidence of Involvement of Bilirubin as Physiological Protector against Oxidative Damage. Biochim Biophys Acta. 1994;1223:9–14. 8061058 10.1016/0167-4889(94)90067-1

[21] Emborg TJ Walker JM Noh B Vierstra RD. Multiple heme oxygenase family members contribute to the biosynthesis of the phytochrome chromophore in Arabidopsis. Plant Physiol. 2006;140:856–868. doi:10.1104/pp.105.074211. 16428602 PMC1400562

[22] Gisk B Yasui Y Kohchi T Frankenberg-Dinkel N. Characterization of the haem oxygenase protein family in Arabidopsis thaliana reveals a diversity of functions. Biochem J. 2010;425:425–434. doi:10.1042/BJ20090775. 19860740

[23] Noriega GO Balestrasse KB Batlle A Tomaro ML. Heme oxygenase exerts a protective role against oxidative stress in soybean leaves. Biochem Biophys Res Commun. 2004;323:1003–1008. doi:10.1016/j.bbrc.2004.08.199. 15381099

[24] Shen Q Jiang M Li H Che LL Yang ZM. Expression of a Brassica napus heme oxygenase confers plant tolerance to mercury toxicity. Plant Cell Environ. 2011;34:752–763. doi:10.1111/j.1365-3040.2011.02279.x. 21241331

[25] Jin Q Zhu K Xie Y Shen W. Heme oxygenase-1 is involved in ascorbic acid-induced alleviation of cadmium toxicity in root tissues of Medicago sativa. Plant Soil. 2013;366:605–616. doi:10.1007/s11104-012-1451-9.

[26] Mahawar L Shekhawat GS. Haem oxygenase: A functionally diverse enzyme of photosynthetic organisms and its role in phytochrome chromophore biosynthesis, cellular signalling and defence mechanisms. Plant Cell Environ. 2018;41:483–500. doi:10.1111/pce.13116. 29220548

[27] Santa-Cruz DM Pacienza NA Polizio AH Balestrasse KB Tomaro ML Yannarelli GG. Nitric oxide synthase-like dependent NO production enhances heme oxygenase up-regulation in ultraviolet-B-irradiated soybean plants. Phytochemistry. 2010;71:1700–1707. doi:10.1016/j.phytochem.2010.07.009. 20708206

[28] Noriega GO Yannarelli GG Balestrasse KB Batlle A Tomaro ML. The effect of nitric oxide on heme oxygenase gene expression in soybean leaves. Planta. 2007;226:1155–1163. doi:10.1007/s00425-007-0561-8. 17569079

[29] Cui W Fu G Wu H Shen W. Cadmium-induced heme oxygenase-1 gene expression is associated with the depletion of glutathione in the roots of Medicago sativa. Biometals. 2011;24:93–103. doi:10.1007/s10534-010-9377-2. 20844928

[30] Yannarelli GG Noriega GO Batlle A Tomaro ML. Heme oxygenase up-regulation in ultraviolet-B irradiated soybean plants involves reactive oxygen species. Planta. 2006;224:1154–1162. doi:10.1007/s00425-006-0297-x. 16703357

[31] Xu S Wang L Zhang B et al RNAi knockdown of rice SE5 gene is sensitive to the herbicide methyl viologen by the down-regulation of antioxidant defense. Plant Mol Biol. 2012;80:219–235. doi:10.1007/s11103-012-9945-7. 22829206

[32] Chen XY Ding X Xu S et al Endogenous hydrogen peroxide plays a positive role in the upregulation of heme oxygenase and acclimation to oxidative stress in wheat seedling leaves. J Integr Plant Biol. 2009;51:951–960. doi:10.1111/j.1744-7909.2009.00869.x. 19778405

[33] Fu GQ Jin QJ Lin YT et al Cloning and characterization of a heme oxygenase-2 gene from alfalfa (Medicago sativa L.). Appl Biochem Biotechnol. 2011;165:1253–1263. doi:10.1007/s12010-011-9343-7. 21870123

[34] Balestrasse KB Yannarelli GG Noriega GO Batlle A Tomaro ML. Heme oxygenase and catalase gene expression in nodules and roots of soybean plants subjected to cadmium stress. Biometals. 2008;21:433–441. doi:10.1007/s10534-008-9132-0. 18228149

[35] Zhang C Li Y Yuan F Hu S He P Effects of hematin and carbon monoxide on the salinity stress responses of Cassia obtusifolia L. Seeds and Seedlings. Plant Soil. 2012;359:85–105. doi:10.1007/s11104-012-1194-7.

[36] Wang L Ma F Xu S et al Cloning and characterization of a heme oxygenase-2 gene from rice (Oryza sativa L.), and its expression analysis in response to some abiotic stresses. Acta Physiol Plant. 2014;36:893–902. doi:10.1007/s11738-013-1468-6.

[37] Muramoto T Kohchi T Yokota A Hwang I Goodman HM. The Arabidopsis photomorphogenic mutant hy1 is deficient in phytochrome chromophore biosynthesis as a result of a mutation in a plastid heme oxygenase. Plant Cell. 1999;11:335–348. 10072395 10.1105/tpc.11.3.335 PMC144190

[38] Izawa T Oikawa T Tokutomi S Okuno K Shimamoto K. Phytochromes confer the photoperiodic control of flowering in rice (a short-day plant). Plant J. 2000;22:391–399. 10849355 10.1046/j.1365-313x.2000.00753.x

[39] Xu DK Jin QJ Xie YJ et al Characterization of a wheat heme oxygenase-1 gene and its responses to different abiotic stresses. Int J Mol Sci. 2011;12:7692–7707. doi:10.3390/ijms12117692. 22174625 PMC3233431

[40] Wang S Zhang KK Huang X Fan YJ Yang LT Li YR. Cloning and functional analysis of thylakoidal ascorbate peroxidase (TAPX) gene in sugarcane. Sugar Tech. 2015;17:356–366. doi:10.1007/s12355-014-0354-x.

[41] Han B Xu S Xie YJ et al ZmHO-1, a maize haem oxygenase-1 gene, plays a role in determining lateral root development. Plant Sci. 2012;184:63–74. doi:10.1016/j.plantsci.2011.12.012. 22284711

[42] Jin QJ Yuan XX Cui WT et al Isolation and characterization of a heme oxygenase-1 gene from Chinese cabbage. Mol Biotechnol. 2012;50:8–17. doi:10.1007/s12033-011-9407-5. 21505948

[43] Gohya T Zhang X Yoshida T Migita CT. Spectroscopic characterization of a higher plant heme oxygenase isoform-1 from Glycine max (soybean)–coordination structure of the heme complex and catabolism of heme. FEBS J. 2006;273:5384–5399. doi:10.1111/j.1742-4658.2006.05531.x. 17076701

[44] Davis SJ Bhoo SH Durski AM Walker JM Vierstra RD. The heme-oxygenase family required for phytochrome chromophore biosynthesis is necessary for proper photomorphogenesis in higher plants. Plant Physiol. 2001;126:656–669. doi:10.1104/pp.126.2.656. 11402195 PMC111157

[45] Krishnamurthy L Serraj R Hash CT Dakheel AJ Reddy BVS Screening sorghum genotypes for salinity tolerant biomass production. Euphytica. 2007;156:15–24. doi:10.1007/s10681-006-9343-9.

[46] Paterson AH. Genomics of sorghum. Int J Plant Genomics. 2008;2008:362451. doi:10.1155/2008/362451. 18483564 PMC2375965

[47] Paterson AH Bowers JE Bruggmann R et al The Sorghum bicolor genome and the diversification of grasses. Nature. 2009;457:551. 19189423 10.1038/nature07723

[48] Mulaudzi-Masuku T Mutepe RD Mukhoro OC Faro A Ndimba B. Identification and characterization of a heat-inducible Hsp70 gene from Sorghum bicolor which confers tolerance to thermal stress. Cell Stress Chaperones. 2015;20:793–804. doi:10.1007/s12192-015-0591-2. 26072391 PMC4529866

[49] Goodstein DM Shu S Howson R et al Phytozome: a comparative platform for green plant genomics. Nucleic Acids Res. 2012;40(Database Issue):D1178–D1186. doi:10.1093/nar/gkr944. 22110026 PMC3245001

[50] Guo AY Hu B Gao G Zhang H Luo J Jin J GSDS 2.0: an upgraded gene feature visualization server. Bioinformatics. 2014;31:1296–1297. doi:10.1093/bioinformatics/btu817. 25504850 PMC4393523

[51] Gasteiger E Hoogland C Gattiker A et al Protein identification and analysis tools on the ExPASy server. In: Walker JM, ed. The Proteomics Protocols Handbook. Totowa, NJ: Humana Press; 2005:571–607. doi:10.1385/1-59259-890-0.

[52] Yu CS Chen YC Lu CH Hwang JK. Prediction of protein subcellular localization. Proteins. 2006;64:643–651. doi:10.1002/prot.21018. 16752418

[53] Larkin MA Blackshields G Brown NP et al Clustal W Clustal X version 2.0. Bioinformatics. 2007;23:2947–2948. doi:10.1093/bioinformatics/btm404. 17846036

[54] Marchler-Bauer A Lu S Anderson JB et al CDD: a Conserved Domain Database for the functional annotation of proteins. Nucleic Acids Res. 2011;39(Database Issue):D225–D229. doi:10.1093/nar/gkq1189. 21109532 PMC3013737

[55] Bailey TL Boden M Buske FA et al MEME Suite: tools for motif discovery and searching. Nucleic Acids Res. 2009;37:W202–208. doi:10.1093/nar/gkp335. 19458158 PMC2703892

[56] Kumar S Stecher G Tamura K. MEGA7: molecular evolutionary genetics analysis version 7.0 for bigger datasets. Mol Biol Evol. 2016;33:1870–1874. doi:10.1093/molbev/msw054. 27004904 PMC8210823

[57] Kao CH. Role of rice heme oxygenase in lateral root formation. Plant Signal Behav. 2013;8:e25766. doi:10.4161/psb.25766. PMC4091076 23887491

[58] Xuan W Zhu FY Xu S et al The heme oxygenase/carbon monoxide system is involved in the auxin-induced cucumber adventitious rooting process. Plant Physiol. 2008;148:881–893. doi:10.1104/pp.108.125567. 18689445 PMC2556841

[59] Shekhawat GS Dixit S Verma K Nasybullina EI Kosmachevskaya OV Topunov a F. Review heme oxygenase : enzyme with functional diversity heme oxygenase: enzyme with functional diversity. J Stress Physiol Biochem. 2011;7:88–94.

[60] Attwood TK. The quest to deduce protein function from sequence: the role of pattern databases. Int J Biochem Cell Biol. 2000;32:139–155. 10687950 10.1016/s1357-2725(99)00106-5

[61] Xie YJ Xu S Han B et al Evidence of Arabidopsis salt acclimation induced by up-regulation of HY1 and the regulatory role of RbohD-derived reactive oxygen species synthesis. Plant J. 2011;66:280–292. doi:10.1111/j.1365-313X.2011.04488.x. 21205037

