# Supplementary material for: Characterization and Expression Analysis of Heme Oxygenase Genes from Sorghum bicolor
Source: Bioinform Biol Insights. 2019 Jul 12;13:1177932219860813. doi: 10.1177/1177932219860813 (PMC6628516; doi:10.1177/1177932219860813)
Supplement: Supplementary_Figure_1_xyz19475d220d457 – Supplemental material for Characterization and Expression Analysis of Heme Oxygenase Genes from Sorghum bicolor [file Supplementary_Figure_1_xyz19475d220d457.pdf]

Supplementary Figure 1.Sequence analysis of the cDNA amplicons of SbHO genes

NB: SbHO1a = SbHO1 Accession numbers: AAK63010.1, AF320026.1

SbHO1b = SbHO4 Accession numbers: XP_021304790.1; XM_021449115.1

SbHO1c = SbHO3 Accession numbers: XP_002438642.1; XM_002438597.2

SbHO2 = SbHO2 Accession numbers: AAK63011.1, AF320027.1

>A12_SbHO1a_purified_PCR_sample_SbHO1a_RV__1

PREDICTED: Sorghum bicolor heme oxygenase 1, chloroplastic (LOC8065066), mRNA
Sequence ID: XM_002438597.2Length: 1237Number of Matches: 1

Alignment statistics for match #1

Score	Expect	Identities	Gaps	Strand
339 bits(183)	3e-89	186/188(99%)	0/188(0%)	Plus/Minus

```
Query  1    GCATATGTAGTGCCAGGGGGCGATGGTTCTGGAATTGTGTGGCCCTGTTGCCTGAACCAT  60
            ||||||||||||||||||||||||||||||||||||||||||||||||||||||||||||
Sbjct  773  GCATATGTAGTGCCAGGGGGCGATGGTTCTGGAATTGTGTGGCCCTGTTGCCTGAACCAT  714

Query  61   TCCAGATCCTTTTTGAGTGCCTCTGATCTCTCCAGCCCAGTGTTCCGGAACTCTGCATAC  120
            ||||||||||||||||||||||||||||||||||||||||||||||||||||||||||||
Sbjct  713  TCCAGATCCTTTTTGAGTGCCTCTGATCTCTCCAGCCCAGTGTTCCGGAACTCTGCATAC  654

Query  121  CACGGGACGGCGGCGCGTTCGACGATGTCTTCGAGCGTCTGGAASACGAGCTTGCTATCG  180
            |||||||||||||||||||||||||||||||||||||||||||| ||||||||||| |||
Sbjct  653  CACGGGACGGCGGCGCGTTCGACGATGTCTTCGAGCGTCTGGAAGACGAGCTTGCTGTCG  594

Query  181  ACAAGGAA  188
            ||||||||
Sbjct  593  ACAAGGAA  586
```

>B12_SbHO1b_purified_PCR_sample_SbHO1b_RV__1

PREDICTED: Sorghum bicolor heme oxygenase 1, chloroplastic (LOC8065066), mRNA
Sequence ID: XM_002438597.2Length: 1237Number of Matches: 1
Related Information

Alignment statistics for match #1

Score	Expect	Identities	Gaps	Strand
307 bits(166)	7e-80	178/184(97%)	2/184(1%)	Plus/Minus

```
Query  5    ATGTAGTG-CAGGAGGCGATGGTTCTGGA-TTGTGTGGCCCTGTTGCCTGAACCATTCCA  62
            |||||||| |||| ||||||||||||||| ||||||||||||||||||||||||||||||
Sbjct  769  ATGTAGTGCCAGGGGGCGATGGTTCTGGAATTGTGTGGCCCTGTTGCCTGAACCATTCCA  710

Query  63   GATCCTTTTTGAGTGCCTCTGATCTCTCCAGCCCAGTGTTCCGGAACTCTGCATACCACG  122
            ||||||||||||||||||||||||||||||||||||||||||||||||||||||||||||
Sbjct  709  GATCCTTTTTGAGTGCCTCTGATCTCTCCAGCCCAGTGTTCCGGAACTCTGCATACCACG  650

Query  123  GGACGGCGGCGCGTKCGACGATGTCTTCGAGCGTCTGGAAGACGAGCTTGCTATCGACGA  182
            |||||||||||||| ||||||||||||||||||||||||||||||||||||| ||||| |
Sbjct  649  GGACGGCGGCGCGTTCGACGATGTCTTCGAGCGTCTGGAAGACGAGCTTGCTGTCGACAA  590

Query  183  GGAA  186
            ||||
Sbjct  589  GGAA  586
```

>C12_SbHO1c_purified_PCR_sample_SbHO1c_RV__1

PREDICTED: Sorghum bicolor heme oxygenase 1, chloroplastic (LOC8065066), mRNA
Sequence ID: XM_002438597.2Length: 1237Number of Matches: 1
Related Information

Alignment statistics for match #1

Score	Expect	Identities	Gaps	Strand
305 bits(165)	2e-79	165/165(100%)	0/165(0%)	Plus/Minus

```
Query  9    AGCATATGTAGTGCCAGGGGGCGATGGTTCTGGAATTGTGTGGCCCTGTTGCCTGAACCA  68
            ||||||||||||||||||||||||||||||||||||||||||||||||||||||||||||
Sbjct  774  AGCATATGTAGTGCCAGGGGGCGATGGTTCTGGAATTGTGTGGCCCTGTTGCCTGAACCA  715

Query  69   TTCCAGATCCTTTTTGAGTGCCTCTGATCTCTCCAGCCCAGTGTTCCGGAACTCTGCATA  128
            ||||||||||||||||||||||||||||||||||||||||||||||||||||||||||||
Sbjct  714  TTCCAGATCCTTTTTGAGTGCCTCTGATCTCTCCAGCCCAGTGTTCCGGAACTCTGCATA  655
```

```
Query  129   CCACGGGACGGCGGCGCGTTCGACGATGTCTTCGAGCGTCTGGAA  173
             |||||||||||||||||||||||||||||||||||||||||||||
Sbjct  654   CCACGGGACGGCGGCGCGTTCGACGATGTCTTCGAGCGTCTGGAA  610
```

>D12_SbHO2_purified_PCR_sample_SbHO2_RV__1

PREDICTED: Sorghum bicolor probable inactive heme oxygenase 2, chloroplastic (LOC8077102), mRNA
Sequence ID: XM 002465125.2Length: 1442Number of Matches: 1

Alignment statistics for match #1

Score	Expect	Identities	Gaps	Strand
396 bits(214)	2e-106	214/214(100%)	0/214(0%)	Plus/Minus

```
Query  11    ATCTTATTGCCTATTGCCACACCTCCAGTTATATGTGCAAAATAGATATTGTAATAATGG  70
             ||||||||||||||||||||||||||||||||||||||||||||||||||||||||||||
Sbjct  791   ATCTTATTGCCTATTGCCACACCTCCAGTTATATGTGCAAAATAGATATTGTAATAATGG  732

Query  71    GAGAGGAATGCAGGAGCGTTGCTTTCAGCCAGTTCAGTCAGATAAGCTGCATAAGTTGAT  130
             ||||||||||||||||||||||||||||||||||||||||||||||||||||||||||||
Sbjct  731   GAGAGGAATGCAGGAGCGTTGCTTTCAGCCAGTTCAGTCAGATAAGCTGCATAAGTTGAT  672

Query  131   CCTGAAGTACTTGGCTCTGGGATTGCGATCCCTTGTTTTCTGAACCACTCCAAATCTTTT  190
             ||||||||||||||||||||||||||||||||||||||||||||||||||||||||||||
Sbjct  671   CCTGAAGTACTTGGCTCTGGGATTGCGATCCCTTGTTTTCTGAACCACTCCAAATCTTTT  612

Query  191   GAAATGCTAGCTGAACGCTCCAAACCACTTTTCC  224
             ||||||||||||||||||||||||||||||||||
Sbjct  611   GAAATGCTAGCTGAACGCTCCAAACCACTTTTCC  578
```

>E11_SbHO1a_purified_PCR_sample_SbHO1a_FW__1

PREDICTED: Sorghum bicolor heme oxygenase 1, chloroplastic (LOC8065066), mRNA
Sequence ID: XM 002438597.2Length: 1237Number of Matches: 1

Alignment statistics for match #1

Score	Expect	Identities	Gaps	Strand
302 bits(163)	3e-78	166/167(99%)	1/167(0%)	Plus/Plus

```
Query  10    CGT-CCGTGGTATGCAGAGTTCCGGAACACTGGGCTGGAGAGATCAGAGGCACTCAAAAA  68
             ||| ||||||||||||||||||||||||||||||||||||||||||||||||||||||||
Sbjct  645   CGTCCCGTGGTATGCAGAGTTCCGGAACACTGGGCTGGAGAGATCAGAGGCACTCAAAAA  704
```

```
Query  69   GGATCTGGAATGGTTCAGGCAACAGGGCCACACAATTCCAGAACCATCGCCCCCTGGCAC  128
            ||||||||||||||||||||||||||||||||||||||||||||||||||||||||||||
Sbjct  705  GGATCTGGAATGGTTCAGGCAACAGGGCCACACAATTCCAGAACCATCGCCCCCTGGCAC  764

Query  129  TACATATGCTTCTCTACTGGAAGAGCTGTCTGAGAAGGATCCCCAGG  175
            |||||||||||||||||||||||||||||||||||||||||||||||
Sbjct  765  TACATATGCTTCTCTACTGGAAGAGCTGTCTGAGAAGGATCCCCAGG  811
```

>F11_SbHO1b_purified_PCR_sample_SbHO1b_FW__1

PREDICTED: Sorghum bicolor heme oxygenase 1, chloroplastic (LOC8065066), mRNA
Sequence ID: XM_002438597.2Length: 1237Number of Matches: 1

Alignment statistics for match #1

Score	Expect	Identities	Gaps	Strand
305 bits(165)	3e-79	170/172(99%)	1/172(0%)	Plus/Plus

```
Query  12   TCGTCG-ACGCGCCGCCGTCCCGTGGTATGCAGAGTTCCGGAACACTGGGCTGGAGAGAT  70
            |||||| |||||||||||||||||||||||||||||||||||||||||||||||||||||
Sbjct  629  TCGTCGAACGCGCCGCCGTCCCGTGGTATGCAGAGTTCCGGAACACTGGGCTGGAGAGAT  688

Query  71   CAGAGGCACTCAAAAAGGATCTGGAATGGTTCAGGCAACAGGGCCACACAATTCCAGAAC  130
            ||||||||||||||||||||||||||||||||||||||||||||||||||||||||||||
Sbjct  689  CAGAGGCACTCAAAAAGGATCTGGAATGGTTCAGGCAACAGGGCCACACAATTCCAGAAC  748

Query  131  CATCGCCCCCTGGCACTACATATGCTTCTCTACTGGAAGAGCTGTCTGGGAA  182
            |||||||||||||||||||||||||||||||||||||||||||||||| |||
Sbjct  749  CATCGCCCCCTGGCACTACATATGCTTCTCTACTGGAAGAGCTGTCTGAGAA  800
```

>G11_SbHO1c_purified_PCR_sample_SbHO1c_FW__1

PREDICTED: Sorghum bicolor heme oxygenase 1, chloroplastic (LOC8065066), mRNA
Sequence ID: XM_002438597.2Length: 1237Number of Matches: 1
Related Information

Alignment statistics for match #1

Score	Expect	Identities	Gaps	Strand
300 bits(162)	1e-77	162/162(100%)	0/162(0%)	Plus/Plus

```
Query  12   CGTGGTATGCAGAGTTCCGGAACACTGGGCTGGAGAGATCAGAGGCACTCAAAAAGGATC  71
            ||||||||||||||||||||||||||||||||||||||||||||||||||||||||||||
Sbjct  650  CGTGGTATGCAGAGTTCCGGAACACTGGGCTGGAGAGATCAGAGGCACTCAAAAAGGATC  709
```

```
Query  72   TGGAATGGTTCAGGCAACAGGGCCACACAATTCCAGAACCATCGCCCCCTGGCACTACAT  131
            ||||||||||||||||||||||||||||||||||||||||||||||||||||||||||||
Sbjct  710  TGGAATGGTTCAGGCAACAGGGCCACACAATTCCAGAACCATCGCCCCCTGGCACTACAT  769

Query  132  ATGCTTCTCTACTGGAAGAGCTGTCTGAGAAGGATCCCCAGG  173
            ||||||||||||||||||||||||||||||||||||||||||
Sbjct  770  ATGCTTCTCTACTGGAAGAGCTGTCTGAGAAGGATCCCCAGG  811
```

>H11_SbHO2_purified_PCR_sample_SbHO2_FW__1

PREDICTED: Sorghum bicolor probable inactive heme oxygenase 2, chloroplastic (LOC8077102), mRNA
Sequence ID: XM_002465125.2Length: 1442Number of Matches: 1

Alignment statistics for match #1

Score	Expect	Identities	Gaps	Strand
375 bits(203)	3e-100	208/210(99%)	1/210(0%)	Plus/Plus

```
Query  13   TTTGGAGTGGTTCAG-ATACAAGGGATCGCAATCCCAGAGCCAAGTACTTCAGGATCAAC  71
            ||||||||||||||| | ||||||||||||||||||||||||||||||||||||||||||
Sbjct  618  TTTGGAGTGGTTCAGAAAACAAGGGATCGCAATCCCAGAGCCAAGTACTTCAGGATCAAC  677

Query  72   TTATGCAGCTTATCTGACTGAACTGGCTGAAAGCAACGCTCCTGCATTCCTCTCCCATTA  131
            ||||||||||||||||||||||||||||||||||||||||||||||||||||||||||||
Sbjct  678  TTATGCAGCTTATCTGACTGAACTGGCTGAAAGCAACGCTCCTGCATTCCTCTCCCATTA  737

Query  132  TTACAATATCTATTTTGCACATATAACTGGAGGTGTGGCAATAGGCAATAAGATCTGCAA  191
            ||||||||||||||||||||||||||||||||||||||||||||||||||||||||||||
Sbjct  738  TTACAATATCTATTTTGCACATATAACTGGAGGTGTGGCAATAGGCAATAAGATCTGCAA  797

Query  192  GAAAATTCTGGAAGGAAGGGAGCTGGAGTT  221
            ||||||||||||||||||||||||||||||
Sbjct  798  GAAAATTCTGGAAGGAAGGGAGCTGGAGTT  827
```
